# Limited evidence for genetic differentiation or adaptation in two amphibian species across replicated rural–urban gradients

**DOI:** 10.1111/eva.13700

**Published:** 2024-06-03

**Authors:** W. Babik, M. Marszałek, K. Dudek, B. Antunes, G. Palomar, B. Zając, A. Taugbøl, M. Pabijan

**Affiliations:** ^1^ Faculty of Biology, Institute of Environmental Sciences Jagiellonian University Kraków Poland; ^2^ Department of Genetics, Physiology and Microbiology, Faculty of Biological Sciences Complutense University of Madrid Madrid Spain; ^3^ Faculty of Biology, Institute of Zoology and Biomedical Research Jagiellonian University Kraków Poland; ^4^ Norwegian Institute for Nature Research Lillehammer Norway

**Keywords:** amphibians, genomics, MHC, urbanization

## Abstract

Urbanization leads to complex environmental changes and poses multiple challenges to organisms. Amphibians are highly susceptible to the effects of urbanization, with land use conversion, habitat destruction, and degradation ranked as the most significant threats. Consequently, amphibians are declining in urban areas, in both population numbers and abundance, however, the effect of urbanization on population genetic parameters remains unclear. Here, we studied the genomic response to urbanization in two widespread European species, the common toad *Bufo bufo* (26 localities, 480 individuals), and the smooth newt *Lissotriton vulgaris* (30 localities, 516 individuals) in three geographic regions: southern and northern Poland and southern Norway. We assessed genome‐wide SNP variation using RADseq (ca. 42 and 552 thousand SNPs in toads and newts, respectively) and adaptively relevant major histocompatibility complex (MHC) class I and II genes. The results linked most of the genetic differentiation in both marker types to regional (latitudinal) effects, which also correspond to historical biogeography. Further, we did not find any association between genetic differentiation and level of urbanization at local scales for either species. However, urban smooth newts, but not toads, have lower levels of within‐population genome‐wide diversity, suggesting higher susceptibility to the negative effects of urbanization. A decreasing level of genetic diversity linked to increasing urbanization was also found for MHC II in smooth newts, while the relationship between MHC class I diversity and urbanization differed between geographic regions. We did not find any effects of urbanization on MHC diversity in the toad populations. Although two genetic environment association analyses of genome‐wide data, LFMM and BayPass, revealed numerous (219 in *B. bufo* and 7040 in *L. vulgaris*) SNPs statistically associated with urbanization, we found a marked lack of repeatability between geographic regions, suggesting a complex and multifaceted response to natural selection elicited by life in the city.

## 1 INTRODUCTION

The extent of densely populated, urban areas of the globe has exploded in recent decades and will continue to grow in the 21st century (Gao & O'Neill, 2020). Natural habitats are negatively impacted by city expansion through a range of factors linked to their general degradation and fragmentation. Urbanization alters the chemical and physical attributes of the environment by increasing the coverage of impervious surfaces (e.g., roads, buildings), which in turn also increase surface temperatures (Yow, 2007), sewage and condensed traffic patterns, decrease the immediate reservoirs of clean water, and elevate pollution levels of air, soil, and water, among others (Grimm et al., 2008). The consequences of urbanization also spill over the fringes of cities into surrounding natural or agricultural habitat. In cities, completely built up areas are usually interwoven with semi‐natural, managed green spaces such as parks, wooded areas, or waterways. The spatial heterogeneity of urban structure and human landscape management produces a rural–urban gradient that encompasses a dense, highly developed core and irregularly spaced, less developed outer perimeters (McDonnell & Pickett, 1990). The ecosystems in this gradient are usually highly disturbed, with variable numbers of native species and typically a large proportion of intentionally or accidentally introduced species (McKinney, 2006, 2008).

Reduction of natural habitat through urbanization is viewed as a major threat to species globally (McKinney, 2002; Simkin et al., 2022). Native species richness tends to decline in more urbanized areas (Aronson et al., 2014; McKinney, 2008) due to strong environmental filtering from a regional to an urban species community (Fournier et al., 2020). Native species that can maintain populations in an urban matrix are often genetically differentiated from rural populations (Johnson & Munshi‐South, 2017). For species that show a high stringency of association to natural habitat or for those that have limited abilities to traverse built‐up areas, fragmentation leads to reduced population size and increased isolation among subpopulations. In consequence, stochastic allele frequency changes due to random genetic drift and founder events become amplified, while gene flow between subpopulations diminishes. In addition, bottlenecks due to direct human impacts (e.g., pollution, persecution) may further reduce population sizes. These processes may decrease genetic diversity within subpopulations and increase genetic differentiation between them (Johnson & Munshi‐South, 2017). These fine‐scale genetic predictions have been recorded for some species (e.g., Delaney et al., 2010), but are not universal (Miles et al., 2019) and should be considered in the context of urban and natural landscape features that restrict or facilitate gene flow in a particular system (Rivkin et al., 2019).

Numerous species exhibit ecological or life history traits enabling them to persist in or even exploit urban environments. Phenotypic changes increasing fitness in cities may result from phenotypic plasticity, simple shifts in frequency of pre‐existing variants or more complex human‐induced eco‐evolutionary feedbacks including the origin of novel traits (Alberti et al., 2017; Johnson & Munshi‐South, 2017; Lambert et al., 2021; Rivkin et al., 2019). Infectious disease ecology in urban wildlife constitutes one particularly important, in terms of human and animal welfare, arena of eco‐evolutionary interactions. Urbanization may recast wildlife‐pathogen interactions through changes in the biology of hosts, pathogens, and vectors (Bradley & Altizer, 2007). For instance, novel urban environments may heighten the risk of exposure of native species to new pathogens and parasites (e.g., Cohen et al., 2022; Rushton et al., 2000), while stress and pollution may induce immunosuppression and increase host susceptibility to infectious diseases (Linzey et al., 2003). Urbanization, through an influence on the immune system, may lead to adaptive genetic divergence in immune genes (Minias, 2023), particularly those directly involved in antigen recognition such as the major histocompatibility complex (MHC) genes (Barnes et al., 2011; DeCandia, Brzeski, et al., 2019; DeCandia, Henger, et al., 2019; Harris et al., 2013; Pikus et al., 2021; Wilbert et al., 2020). Exceptionally high variability and propensity for rapid evolution make genes coding for molecules involved in interactions with pathogens important candidates for urban adaptation.

Amphibians are highly threatened at the global level, with land use conversion, habitat destruction, and degradation ranked as the most significant contributors (Cordier et al., 2021; Luedtke et al., 2023). Amphibians are highly susceptible to the effects of urbanization (Cordier et al., 2021; Hamer & McDonnell, 2008; Mitchell & Brown, 2008). Impervious surfaces and especially roads in built‐up areas constitute formidable barriers to dispersal for many amphibian species, which are small‐sized and relatively sedentary (Andrews et al., 2008). An ectothermic physiology and highly permeable skin render them vulnerable to altered thermal regimes, chemical agents, and pollutants (Wells, 2019). Moreover, many amphibians exhibit a biphasic life cycle in which larvae require clean, freshwater habitats, while post‐metamorphic stages move into moist terrestrial environments such as forest or wet meadows, but return to water for breeding. Transitions between terrestrial and aquatic environments necessitate the existence of both habitat types in proximity without migration barriers (Hamer & McDonnell, 2008; Semlitsch & Bodie, 2003). These ecological requirements, fundamental for sustaining viable amphibian populations, may be difficult to achieve in urban areas. Other important drivers of amphibian decline include pollution (Walsh et al., 2005), spread of infectious disease (Carey et al., 2003) and introduction of exotic and predatory fish, crayfish and frog species (reviewed in Hamer & McDonnell, 2008), all of which can be exasperated in cities (Mitchell & Brown, 2008). Further threats to amphibians associated with urbanized areas include road kills (Hamer et al., 2015) and ecological traps, for example, stormwater pools replacing natural wetlands in and around cities (Sievers et al., 2018).

Given these physiological and ecological limitations, it is unsurprising that amphibian species richness and abundance decline in conjunction with urbanization (Callaghan et al., 2021; Hamer & McDonnell, 2008; Scheffers & Paszkowski, 2012). However, evidence for a negative influence of urbanization on genetic diversity of amphibian populations is mixed. Although many studies have reported stronger differentiation and/or erosion of genetic variability in urban populations (Arens et al., 2007; de Campos Telles et al., 2007; Fusco et al., 2021; Hitchings & Beebee, 1997, 1998; Homola, Loftin, & Kinnison, 2019; Lourenço et al., 2017; Munshi‐South et al., 2013; Noël et al., 2007; Noël & Lapointe, 2010; Vargová et al., 2023), others have found no or only a weak effect (Furman et al., 2016; Jehle et al., 2023; Schmidt & Garroway, 2021; Straub et al., 2015; Yannic et al., 2021). Schmidt and Garroway (2021) provided a synthesis for 19 North American amphibian species across rural–urban gradients and did not detect a relationship between genetic parameters and urbanization. They argued that the response of amphibian populations to urbanization is not amenable to generalization but instead is species‐specific and contingent on variation in local environmental variables (see also Rivkin et al., 2019). However, most previous studies were based on genetic variation in a handful of microsatellite loci, and the results (particularly a lack of effect) may be a function of the number and resolution of the applied molecular markers, and not population history (McCartney‐Melstad et al., 2018).

**FIGURE 1:**
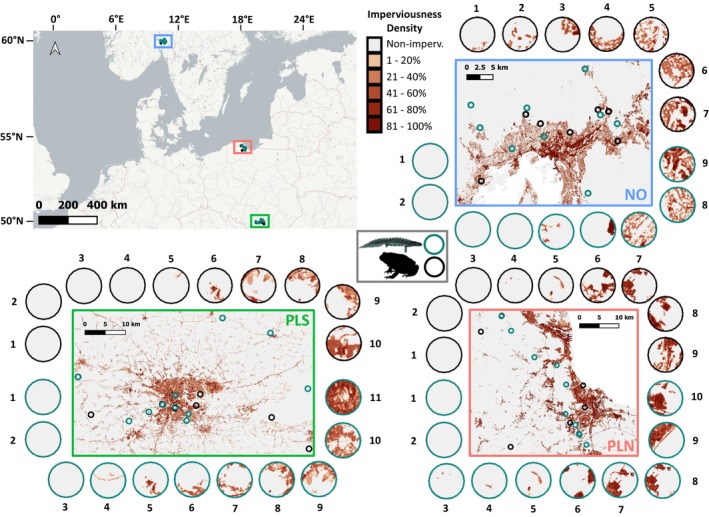
Sampling. Circles show the distribution of surface imperviousness within a radius of 1 km from ponds. Note that within each region localities of each species are numbered independently.

Here, our aim was to assess the effects of urbanization on putative neutral and adaptive variation in two amphibian species using replicated rural–urban sampling along a latitudinal gradient in Europe. We employed thousands of SNP loci and genotyped highly variable MHC class I (MHC‐I) and class II (MHC‐II) to assess genetic structure across the rural–urban gradient and test for associations between genetic diversity measures and levels of urbanization. Using genetic environment association (GEA) analyses, we examined whether parallel signals of urban–rural differentiation, indicative of a potential adaptive response to the urban environment, are detectable at the level of individual SNPs. We focused on two European amphibian species: an anuran, the common toad (*Bufo bufo*), and a urodele, the smooth newt (*Lissotriton vulgaris*). Although declining locally in some areas (e.g., Carrier & Beebee, 2003; Sinsch et al., 2018), both species inhabit a broad range of habitats including urban areas (e.g., Kaczmarski et al., 2020), and have Least Concern IUCN status. Their wide distributions and presumed large populations, signaling ample standing genetic variation, make them ideal candidates for studying the effects of drift and selection in urban settings. We predicted that if urban habitat affects allele frequencies in populations of these two amphibian species, then the genomic signal of urbanization should be reiterated across replicate geographic regions with similar urban structure (Figure 1).

## 2 MATERIALS AND METHODS

### 2.1 Samples

The common toad (*Bufo bufo*) and the smooth newt (*Lissotriton vulgaris*) were sampled with nets and traps during three consecutive breeding seasons (2021–2023) from multiple localities forming urbanization gradients in three widely spaced geographic regions (Figure 1): southern Norway (the area of Oslo, NO), northern Poland (the area of Gdańsk, PL N), and southern Poland (the area of Kraków, PL S). There is evidence that at least one of our sampling regions, PL S, contains urban water bodies with higher levels of chemical runoff and lower amphibian species richness (Budzik et al., 2014). Toe (*B. bufo*) and tailtip (*L. vulgaris*) biopsies were preserved in 96% ethanol and the animals were immediately released. Altogether, we sampled 26 *B. bufo* and 30 *L. vulgaris* pond‐breeding populations (Table 1).

**TABLE 1:** Sampling and basic variation statistics.

Species	Locality	Region	Lat	Lon	Urb. score	N RAD	Mean N	Hs	N MHCI	N MHCII	Nall MHCI	Nall MHCII	AR MHCI	AR MHCII	NAllind MHCI	NAllind MHCII
*Bufo bufo*	Bb_NO_1	NO	59.965	10.706	1.2	21	19.6	0.208	21	17	30	3	26	3	11.6	1.6
*B. bufo*	Bb_NO_2	NO	59.981	10.656	4.8	20	18.7	0.194	20	19	31	3	25.4	3	11.3	1.7
*B. bufo*	Bb_NO_3	NO	59.989	10.901	10.2	20	17.7	0.212	20	19	35	4	28	3.5	11.7	1.6
*B. bufo*	Bb_NO_4	NO	59.950	10.806	11.8	19	17.1	0.189	19	19	28	4	23.1	3.1	10.9	1.4
*B. bufo*	Bb_NO_5	NO	59.866	10.505	13.0	6	5.6	0.194	9	8	31	3	29.6	3	12.6	1.5
*B. bufo*	Bb_NO_6	NO	59.935	10.968	19.2	13	12.1	0.205	15	10	29	3	24.8	3	10.9	1.4
*B. bufo*	Bb_NO_7	NO	59.986	10.937	25.0	20	17.8	0.211	20	20	39	4	30.7	3.4	11.8	1.4
*B. bufo*	Average/Total	NO	NA	NA	NA	17	15.5	0.202	17.7	16	68	5	26.8	3.1	11.5	1.5
*B. bufo*	Bb_PLN_1	PL N	54.277	18.321	0.0	15	13.2	0.242	15	15	39	4	29.7	3.6	10.1	1.6
*B. bufo*	Bb_PLN_2	PL N	54.539	18.205	0.0	20	17.6	0.255	20	20	46	6	28.1	4.4	10	1.7
*B. bufo*	Bb_PLN_3	PL N	54.575	18.286	0.0	20	17.7	0.253	20	20	48	7	30.1	5.4	10.4	1.9
*B. bufo*	Bb_PLN_4	PL N	54.481	18.414	0.3	16	13.8	0.247	16	16	40	5	29.9	4.5	10.6	2
*B. bufo*	Bb_PLN_5	PL N	54.419	18.538	1.8	18	15.8	0.244	18	18	44	4	29.3	3.8	9.9	1.7
*B. bufo*	Bb_PLN_6	PL N	54.332	18.553	17.1	19	16.8	0.239	19	19	50	6	31.8	4.1	10.2	1.6
*B. bufo*	Bb_PLN_7	PL N	54.305	18.590	17.4	20	17.5	0.237	20	20	38	4	28.2	4	10.9	2
*B. bufo*	Bb_PLN_8	PL N	54.417	18.604	18.8	19	16.6	0.240	20	20	33	5	25.5	4.4	10.4	2
*B. bufo*	Bb_PLN_9	PL N	54.367	18.611	20.5	20	17.4	0.237	20	20	41	4	29.7	3.8	10.7	1.8
*B. bufo*	Average/Total	PL N	NA	NA	NA	18.6	16.3	0.244	18.7	18.7	126	9	29.1	4.2	10.4	1.8
*B. bufo*	Bb_PLS_1	PL S	50.013	20.287	0.0	17	14.9	0.258	17	17	54	11	35	7.7	11.1	2.3
*B. bufo*	Bb_PLS_2	PL S	50.076	20.411	0.0	20	17.4	0.260	20	20	69	10	41	6.7	11.6	1.9
*B. bufo*	Bb_PLS_3	PL S	50.019	19.669	0.0	20	16.7	0.256	20	20	63	6	41.2	5.3	11.8	2.5
*B. bufo*	Bb_PLS_4	PL S	50.102	19.625	0.0	20	17.3	0.260	20	20	63	6	36.8	4.7	10.9	2
*B. bufo*	Bb_PLS_5	PL S	49.944	20.415	0.4	13	11.1	0.253	13	13	53	7	39.4	6.5	11.5	1.9
*B. bufo*	Bb_PLS_6	PL S	50.025	19.867	4.0	24	21.1	0.260	24	24	66	7	37.2	4.3	11.2	1.7
*B. bufo*	Bb_PLS_7	PL S	50.034	19.956	8.7	20	17.5	0.248	20	20	33	7	25.1	5.8	10.2	2.1
*B. bufo*	Bb_PLS_8	PL S	50.042	19.913	9.0	20	17.5	0.253	20	20	47	7	29.5	5.5	10.1	1.9
*B. bufo*	Bb_PLS_9	PL S	50.064	20.043	11.4	20	17.2	0.251	20	20	47	7	31.9	4.8	11.2	2
*B. bufo*	Bb_PLS_10	PL S	50.039	20.029	23.7	20	17.1	0.250	20	20	44	8	31.2	4.7	11.2	1.8
*B. bufo*	Average/Total	PL S	NA	NA	NA	19.4	16.8	0.255	19.4	19.4	197	16	34.8	5.6	11.1	2
*Lissotriton vulgaris*	Lv_NO_1	NO	59.992	10.660	0.0	23	18	0.169	23	23	30	10	27	7.5	12	2.1
*L. vulgaris*	Lv_NO_2	NO	59.997	10.470	0.0	3	2.6	0.130	3	3	23	4	NA	NA	12	3
*L. vulgaris*	Lv_NO_3	NO	59.965	10.966	0.0	16	13	0.189	16	16	34	8	29.4	7.1	12.6	2.4
*L. vulgaris*	Lv_NO_4	NO	59.846	10.867	2.0	20	16.3	0.182	20	20	37	7	31.6	6.3	12.1	2.6
*L. vulgaris*	Lv_NO_5	NO	59.958	10.501	2.1	16	12.9	0.164	17	17	35	8	29	6.1	12.4	2.1
*L. vulgaris*	Lv_NO_6	NO	59.980	10.909	9.6	13	10.6	0.183	14	14	39	11	32.8	9	11.6	2.6
*L. vulgaris*	Lv_NO_7	NO	59.922	10.609	14.1	13	11	0.173	14	14	34	6	31.4	5.7	12.6	2.4
*L. vulgaris*	Lv_NO_8	NO	60.059	10.857	15.7	21	17.4	0.187	23	23	43	4	33.7	4	13.4	1.9
*L. vulgaris*	Lv_NO_9	NO	59.943	10.718	31.6	18	14.2	0.139	18	18	34	4	30.8	4	14.4	2.4
*L. vulgaris*	Average/Total	NO	NA	NA	NA	15.9	12.9	0.168	16.4	16.4	69	16	30.7	6.2	12.6	2.4
*L. vulgaris*	Lv_PLN_1	PL N	54.282	18.617	0.0	18	13.9	0.184	18	18	45	14	36.3	9.6	14	2.9
*L. vulgaris*	Lv_PLN_2	PL N	54.574	18.285	0.0	16	12.6	0.186	23	23	54	11	41.3	8.2	13	2.7
*L. vulgaris*	Lv_PLN_3	PL N	54.481	18.414	0.3	17	12.5	0.188	17	17	49	9	38.9	8.1	12.8	2.7
*L. vulgaris*	Lv_PLN_4	PL N	54.541	18.323	0.4	20	16.7	0.191	20	20	49	11	36.4	8.8	12.7	3
*L. vulgaris*	Lv_PLN_5	PL N	54.419	18.538	1.8	20	17.1	0.190	20	20	49	10	39	8.7	12.7	2.8
*L. vulgaris*	Lv_PLN_6	PL N	54.327	18.574	12.2	16	12.1	0.188	16	16	48	9	38	7.3	12.1	3
*L. vulgaris*	Lv_PLN_7	PL N	54.307	18.587	16.6	16	12.4	0.187	16	16	46	9	37.5	7.3	13.2	2.2
*L. vulgaris*	Lv_PLN_8	PL N	54.305	18.59	17.4	20	14.7	0.186	20	20	44	11	34.5	7.8	12.6	3
*L. vulgaris*	Lv_PLN_9	PL N	54.463	18.501	23.0	18	14.7	0.193	18	18	50	14	39.9	9.4	12.7	2.6
*L. vulgaris*	Lv_PLN_10	PL N	54.352	18.535	23.8	18	13.1	0.183	18	18	48	13	40.5	9.9	12.6	3.4
*L. vulgaris*	Average/Total	PL N	NA	NA	NA	17.9	14	0.188	18.6	18.6	80	27	38.2	8.5	12.8	2.8
*L. vulgaris*	Lv_PLS_1	PL S	50.076	20.411	0.0	16	11.3	0.197	16	16	51	18	39	13.4	12.6	3.2
*L. vulgaris*	Lv_PLS_2	PL S	50.102	19.624	0.0	18	15.3	0.205	18	18	46	14	34.4	12	12.4	3.5
*L. vulgaris*	Lv_PLS_3	PL S	50.005	19.799	0.0	10	8.4	0.204	10	10	47	15	43.3	13	12.7	3
*L. vulgaris*	Lv_PLS_4	PL S	50.231	20.119	0.9	15	12	0.203	15	15	50	12	38.6	10.6	13.5	3.4
*L. vulgaris*	Lv_PLS_5	PL S	50.025	19.867	4.0	15	10.5	0.196	15	15	45	13	37.1	10.9	12.7	3.3
*L. vulgaris*	Lv_PLS_6	PL S	50.198	20.283	5.1	16	13	0.204	16	16	59	14	44.7	10.3	13.2	3.2
*L. vulgaris*	Lv_PLS_7	PL S	50.006	19.996	8.1	20	14.4	0.202	20	20	50	17	36.4	11.4	12.4	3.3
*L. vulgaris*	Lv_PLS_8	PL S	50.041	19.915	11.4	20	17.1	0.213	20	20	58	18	42.1	11.9	12.3	3.1
*L. vulgaris*	Lv_PLS_9	PL S	50.036	19.956	12.0	20	16.5	0.210	20	20	59	16	42	11.5	12.6	3.4
*L. vulgaris*	Lv_PLS_10	PL S	50.021	20.002	20.6	24	16.2	0.180	24	24	48	10	33.7	8.1	13.1	3.7
*L. vulgaris*	Lv_PLS_11	PL S	50.062	19.957	47.4	20	14.6	0.174	20	20	30	11	26.4	8.3	12.2	3.4
*L. vulgaris*	Average/Total	PL S	NA	NA	NA	17.6	13.6	0.199	17.6	17.6	105	38	38	11	12.7	3.3

Abbreviations: AR, allelic richness, the number of alleles per locality standardized to the sample size of 8; Hs, expected heterozygosity; mean *N*, mean sample size per SNP, as calculated by Stacks *populations*; N MHCI, N MHCII, the number of individuals scored in MHC‐I and MHC‐II, respectively; N RAD, the number of individual analyzed with RADseq; NA, not applicable; Nall, number of alleles per locality; NAllind, mean number of alleles per individual; NO, Norway (the area of Oslo); PL N, northern Poland (the area of Gdańsk); PL S, southern Poland (the area of Kraków); Urb score, urbanization score, percentage of impervious surface within 1 km of the site.

### 2.2 Measuring urbanization

We calculated an urbanization score for each population by extracting the average percentage of impervious surface from the high‐resolution layer database of the European Environment Agency (https://land.copernicus.eu/pan‐european) in a 1‐km buffer around each locality using Quantum‐GIS (Team, 2016). The 1‐km buffer zone was selected to approximately reflect the likely dispersal capacity of both species.

### 2.3 Laboratory procedures

#### 2.3.1 RADseq

DNA was extracted using the Wizard Genomic DNA Purification Kit (Promega). Double digest RADseq libraries were prepared according to the Adapterama III High‐Throughput 3RAD protocol (Bayona‐Vásquez et al., 2019) from 100 ng of genomic DNA, using restriction enzymes *Eco*RI, *Xba*I, and *Nhe*I. Fragments in the range of 490–600 bp were excised using Pippin Prep, the libraries were pooled equimolarly and sequenced (2 × 150 bp) by Novogene on a NovaSeq 6000 instrument. Replicate libraries were prepared and sequenced for 36 *B. bufo* and 39 *L. vulgaris* samples to estimate the genotyping error.

#### 2.3.2 MHC

To amplify a 210 bp fragment of the third exon of MHC‐I in *B. bufo* two forward (F1: CTGTGAGMTGARAGATGAYG, F2: CTGTGAGCRGAGAGATGRCG) and one reverse (R: TCTCCKCTCYAGATCTTCTC) primers were designed. Primers described in Zeisset and Beebee (2013) were used to amplify a 282 bp fragment of the second exon of MHC‐II. In *L. vulgaris*, MHC‐I was amplified as described in detail in Fijarczyk et al. (2018) and MHC‐II as described in Dudek et al. (2019). For both species, MHC fragments were amplified in 10 μL PCR reactions containing: 50–100 ng of genomic DNA, 5 uL of Multiplex PCR kit (Qiagen) and primers at concentrations of 0.5–1 uM. Individuals were barcoded with a combination of 6 bp indexes at the 5′ end of forward and reverse primers. PCR conditions for MHC‐I amplification were: initial denaturation at 95°C for 15 min, followed by 33 cycles: 95°C for 30 s, 56°C for 30 s and 72°C for 70 s, and final elongation at 72°C for 10 min. PCR conditions for MHC‐II amplification were: initial denaturation at 95°C for 15 min, followed by 35 cycles: 95°C for 30 s, 55°C for 30 s and 72°C for 70 s, and final elongation at 72°C for 10 min. Amplicons were pooled approximately equimolarly based on gel‐band intensity, pools were gel‐purified, Illumina adaptors were ligated using NEBNext Ultra II DNA Library Prep Kit for Illumina (New England Biolabs) according to the manufacturer's protocol optimized for a PCR‐free workflow. Libraries were sequenced on an Illumina MiSeq using v2 500 cycles kits. To estimate the MHC genotyping error, 4 (MHC‐I) and 5 (MHC‐II) *B. bufo* as well as 17 (for each MHC class) *L. vulgaris* samples were amplified, sequenced and genotyped in replicates.

### 2.4 Bioinformatics

#### 2.4.1 RADseq

Reads were demultiplexed and cleaned with *process_radtags* from Stacks 2.64 (Rochette et al., 2019) using parameters ‐*q* (discard reads with low quality scores), and ‐*r* (rescue barcodes and RAD‐tag cut sites). Presently, a reference genome is only available for *B. bufo*, and subsequent steps were, therefore, performed differently for each species.

The *B. bufo* reads were mapped to the reference genome aBufBuf1.1 (GCF_905171765.1) with Bowtie2 2.4.2 using default settings. The resulting bam files were further processed with *gstacks*, with increased stringency for discovering SNPs (‐‐var‐alpha 0.001) and calling genotypes (‐‐gt‐alpha 0.01), in addition to removing unpaired reads (‐‐rm‐unpaired‐reads).

For *L. vulgaris* we used the Stacks de novo assembly procedure. First, we identified the optimal values of three key assembly parameters: *M* (distance allowed between stacks), *m* (minimum stacks depth), and *n* (distance allowed between catalogue loci). To this end, we used the approach of Paris et al. (2017) and Rochette and Catchen (2017), which identifies the combination of the parameters that maximizes the number of RAD loci present in a minimum of 80% samples. This procedure was performed with 18 samples, six per geographic region, sequenced to a similar depth (ca. 6.5 million read pairs). We initially tested the *M* values in the range of 2–6, *m* = 3 or 5 and *n* = *M* or *n* = (*M* + 2). Following the preliminary tests, we also examined *M* in the range of 7–10 and *n* = *M* or *n* = (*M* + 2) for *m* = 3 only. The values *M* = 7, *m* = 3 and *n* = 9 were identified as optimal and used in all further analyses. Following identification of RAD loci within individuals with *ustacks*, the catalog of RAD loci was created with *cstacks* from 75 samples (25 per region, ca. 7.5 million read pairs per sample) to reduce the computational burden. Loci from all samples were matched to the catalog loci with *sstacks*, the resulting tab‐delimited files were converted to .bam files with tsv2bam, and *gstacks* was run on the .bam files with ‐‐var‐alpha 0.001 and ‐‐gt‐alpha 0.01.

The results of *gstacks* were further processed in *populations* and with custom bash and R scripts with identical settings for both species. In *populations*, we retained RADloci present in at least 50% of individuals overall (−*R* 0.5), and when applying these filters haplotype‐wise (‐*H*); only biallelic SNPs with a global minor allele frequency (MAF) of at least 0.02 (‐‐min‐maf 0.02) were retained. For each SNP in each population, the *p‐* value of the two‐sided test of Hardy–Weinberg proportions and *F*_IS_ were also calculated. The blacklist of RADloci to be excluded from further analyses because of an excess of heterozygosity (which suggests collapsed paralogs) or an extreme excess of homozygosity was compiled as follows. First, for each locus in each population, the SNP with the lowest HWE *p* value was identified. Then, loci were blacklisted if they showed: (1) heterozygote excess (*p* < 0.01) in more than one population, (2) heterozygote deficit (*p* < 0.01) in more than half of the polymorphic populations with a genotyped sample size of 8. Populations was run again with the compiled blacklist, and in the case of *B. bufo* the resulting .vcf file was further filtered with bcftools 1.9 (Danecek et al., 2021) to retain only chromosomal SNPs (SNPs on scaffolds not assigned to chromosomes were discarded). Genotypes with quality below 20 or coverage less than 8 were set to missing, and only SNPs with less than 50% missing data were retained for subsequent analyses.

#### 2.4.2 MHC

MHC genotyping was accomplished using the adjustable clustering method implemented in AmpliSAS (Sebastian et al., 2016). Bioinformatics procedures used for MHC‐I genotyping followed the protocol described in Fijarczyk et al. (2018), and for MHC‐II, the protocol described in Dudek et al. (2019) was applied.

### 2.5 Genetic variation, geographic structure and tests for the effect of urbanization

The overall genetic structure in each species was assessed with several complementary methods. Principal component analysis (PCA) was conducted in plink 1.9 (Chang et al., 2015) on linkage disequilibrium (LD)‐pruned data (‐‐indep 50 5 2). The relationships between populations were reconstructed with Treemix 1.13 (Pickrell & Pritchard, 2012) for which we selected six (*B. bufo*) or eight (*L. vulgaris*) individuals from each population with the least missing data. To identify the number of genetic clusters (*K*) present in each dataset, we ran Admixture 1.3 (Alexander et al., 2009) on LD‐pruned data; we evaluated *K* from 1 to 10 and identified the most likely value of *K* as the one minimizing the cross‐validation error (CVE). The matrix of pairwise *F*_ST_ between populations based on the presence/absence of MHC alleles was calculated for each species and MHC class separately, using function pairwise.fst.dosage() from the R package hierfstat (Goudet et al., 2015) and relationships between localities were visualized with Multidimensional Scaling.

The potential effect of urbanization on genetic variation was tested with linear models for the following dependent variables: (i) average expected SNP heterozygosity, (ii) MHC allelic richness (each MHC class separately), and (iii) mean number of MHC alleles per individual (each MHC class separately). As explanatory variables we used the urbanization score, region, and their interaction. To identify MHC alleles with significant frequency shifts along the urbanization gradient, we fitted generalized linear models (family binomial) with allele presence/absence as the dependent variable. Urbanization score, region, and their interaction were used as fixed factors while population was included as a random factor nested within region. Only MHC alleles occurring in at least 20% of individuals in at least two regions were tested. The models were fit using lm() and glmer() in R and the significance of main effects and interaction was calculated using Anova() from the car package (Fox & Weisberg, 2019) in R with type III SS. Bonferroni correction was applied for multiple testing. We assessed the spatial autocorrelation of the residuals of our models using Moran's *I* test. To do this, we first defined spatial neighbors using a Gabriel graph, and then transformed it to compute a spatially weighted matrix that weighted edges as a function of geographic distance. This was done using the R package spdep (Pebesma & Bivand, 2023).

We used two methods to identify markers that respond to urbanization while controlling for confounders due to overall genetic differentiation: Latent Factors Mixed Models (LFMM, Caye et al., 2019) as implemented in function lfmm2() from the R package LEA (Gain & François, 2021), and BayPass (Gautier, 2015). Each species was analyzed separately. Both methods were run for SNPs with MAF ≥0.05. Because the LFMM method cannot handle missing data, we imputed missing genotypes with impute() from LEA under the assumption of *K* = 3 genetic clusters. We also used three latent factors (*K* = 3) in lfmm2(). The *p*‐values were calculated with lfmm2.test() from LEA, where the *p*‐values for SNPs from putative collinear and inversion regions were combined, and the false discovery rate (FDR) was corrected using the *p*.adjust() R function with the method “fdr”. In BayPass 2.31 we tested for associations between individual SNPs and urbanization using the auxiliary variable covariate (AUX) model, providing the population covariance matrix Ω calculated under the core model (Gautier, 2015). The median Bayes Factor value (in decibans [dB = 10log_10_BF]) was calculated as the median from five independent BayPass runs, and a value >20 was considered as “decisive” evidence for an association (Gautier, 2015). In addition to the analyses based on the full dataset for both species, we also ran LFMM and BayPass for each geographic region separately to assess consistency between regions and to detect possible region‐specific signals.

### 2.6 Genes associated with urbanization candidates

To determine whether urbanization candidates identified by LFMM or BayPass are associated with protein coding genes or their specific functional categories, we performed two analyses. First, we tested whether urbanization candidates were more often than other markers found within protein coding sequences (CDS) or were associated with protein coding genes. Second, we tested whether any gene ontology (GO) categories were overrepresented among genes associated with urbanization candidates. For *B. bufo* we used the available genome annotation. As no *L. vulgaris* genome assembly is available, we used the genome assembly of *Pleurodeles waltl* (Brown et al., 2022), another newt species with approximately 50 my divergence from *L. vulgaris* (timetree.org). We mapped sequences of *L. vulgaris* RAD loci that were used in LFMM and BayPass analyses to the *P. waltl* genome using minimap2 (Li, 2018) with settings appropriate for divergent sequences that may contain large gaps, as expected for some RAD loci: ‐x map‐ont ‐‐splice ‐g1k ‐G1k ‐A1 ‐B2 ‐O2,32 ‐E1,0 ‐un ‐N2. We then kept only primary alignments with mapping quality >30, indicating unambiguous mapping to a single genomic location.

We then checked, for each *B. bufo* SNP and each mapped *L. vulgaris* RAD locus, using *bedtools closest*: (1) whether it overlapped a CDS and (2) the distance to the closest annotated gene—we considered a SNP/locus to be associated with a gene if it overlapped an annotated gene (including introns and untranslated regions) or was placed less than 10 kb away from a gene. We tested whether urbanization candidates overlapped CDSs or were associated with genes more frequently than expected by chance using the chi‐squared test (R function chisq.test()). The GO terms were assigned to *B. bufo* and *P. waltl* genes using predicted protein sequences and eggNOG‐mapper v. 2 (Cantalapiedra et al., 2021). We then tested for overrepresentation of GO terms among genes associated with urbanization candidates using the R package topGO (Alexa & Rahnenfuhrer, 2022), applying Fisher's exact test and the “weight01” algorithm (Alexa et al., 2006) to deal with the GO graph structure; only GO categories with at least 10 members were considered.

## 3 RESULTS

### 3.1 Genetic diversity and geographic structuring

We obtained on average 4.4 ± (SD) 2.5 (*Bufo bufo*) and 6.4 ± 2.3 (*Lissotriton vulgaris*) million read pairs per individual, of which 99 and 92%, respectively, were identified as valid RADseq reads. After the initial filtering steps, the RADseq datasets contained 41,819 SNPs at 16,294 RAD loci in 480 *B. bufo* individuals from 26 localities, and 552,085 SNPs at 50,477 RAD loci in 516 *L. vulgaris* individuals from 30 localities (Table 1, Figure 1). The fraction of missing genotypes was 15.6% in *B. bufo* and 29.8% in *L. vulgaris*, and the genotyping error, measured as the Non‐Reference Discordance, was 2.6% and 5.0%, respectively. The highly variable 2nd exon of MHC genes was genotyped in the same populations and mostly the same individuals as RADseq. MHC‐I was genotyped in 497 *B. bufo* and 528 *L. vulgaris*, while MHC‐II in 477 *B. bufo* and 528 *L. vulgaris* individuals (Table 1). In MHC‐I we detected a total of 300 alleles in *B. bufo* and 158 in *L. vulgaris*, while in MHC‐II a total of 21 alleles in *B. bufo* and 59 in *L. vulgaris*. The repeatability of genotyping in *B. bufo* was 100% for both MHC classes, while in *L. vulgaris* it was 96% for MHC‐I and 95% for MHC‐II.

**FIGURE 2:**
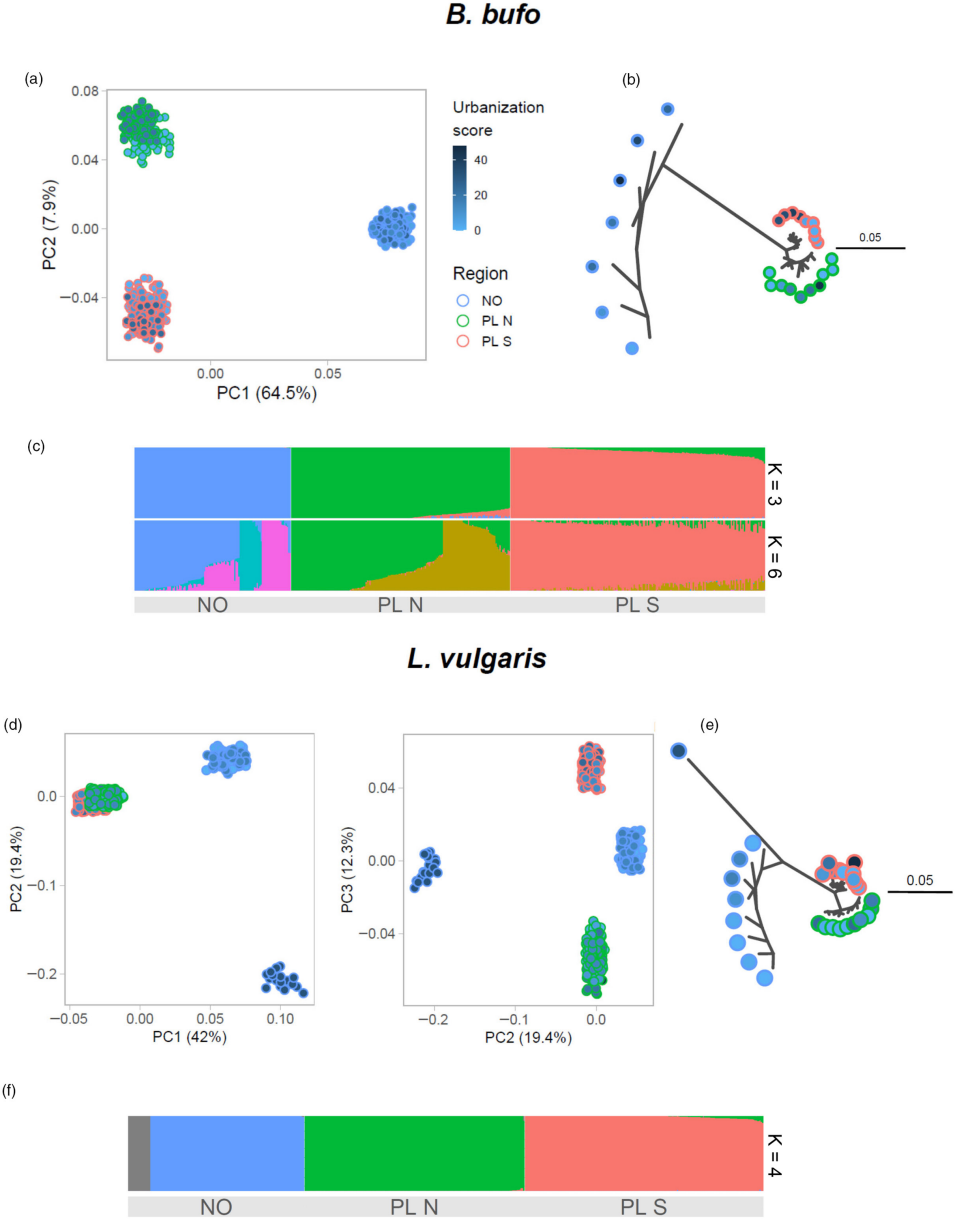
Genetic differentiation – RADseq SNP data. (a, d) Principal component analysis results; note that some jitter was introduced to better visualize points; (b, e) Treemix drift trees, branch lengths reflect the amount of drift specific to a particular branch; (c, f) Admixture results, with the assumed number of genetic clusters (*K*) indicated on the right side of the plots. The fill scale of individual (a, d) and population (b, e) shows urbanization score – the same scale was used for both species.

When comparing genetic variation for *B. bufo* between the three geographic regions, the PCA showed a clear differentiation between Poland and Norway along the first axis (PC1), and separated the three regions, in particular PL N and PL S, along the second axis (PC2). The second PCaxis explained eight times less variation than PC1 (Figure 2a), a trend that was also evident from the Treemix analysis, suggesting a higher rate of population‐specific drift in Norway (Figure 2b). The Admixture analysis supported *K* = 6 genetic clusters, with a within‐region sub‐structure visible in Norway and northern Poland (Figure 2c). However, a small difference in CVE between *K* = 6 and *K* = 3 (CVE_6_ = 0.456 vs. CVE_3_ = 0.461) indicates considerable support also for three genetic clusters.

When comparing genetic variation in *L. vulgaris*, PC1 also separated Norwegian from Polish populations (Figure 2d). However, along PC2, a single Norwegian population, Lv_NO_9, was identified as being genetically distinct from all other samples (Figure 2d). The Treemix drift tree clearly showed that the divergence of Lv_NO_9 was the result of extremely strong population‐specific drift (Figure 2e). Otherwise, Treemix showed differentiation between the three geographic regions, with more population‐specific drift in the Norwegian populations and relatively low divergence between PL N and PL S. In the admixture analysis *K* = 4, with Lv_NO_9 assigned to a separate cluster (Figure 2f) was supported over *K* = 3 (CVE_4_ = 0.429 vs. CVE_3_ = 0.456). Notably, population Lv_NO_9 also showed the lowest heterozygosity of all the investigated populations (except one population with a very small sample size of 3, Table 1).

MHC differentiation between regions was visible in frequencies of individual alleles (Figure S1) and in the multidimensional scaling of the MHC *F*_ST_ matrices for both species (Figure S2). The latter suggests stronger or more easily detectable differentiation in MHC‐I.

### 3.2 The effect of urbanization on genetic variation

Urbanization scores formed a gradient with the range 0.0–25.0 in *B. bufo* and 0.0–47.4 in *L. vulgaris* (Figures 1 and S3, Table 1). We did not find evidence for increased genetic differentiation with increasing urbanization score for either *B*. *bufo* or *L. vulgaris*, or any signs of higher rates of genetic drift in populations with high values of urbanization score, which would have been manifested as longer branches in the Treemix analysis.

**FIGURE 3:**
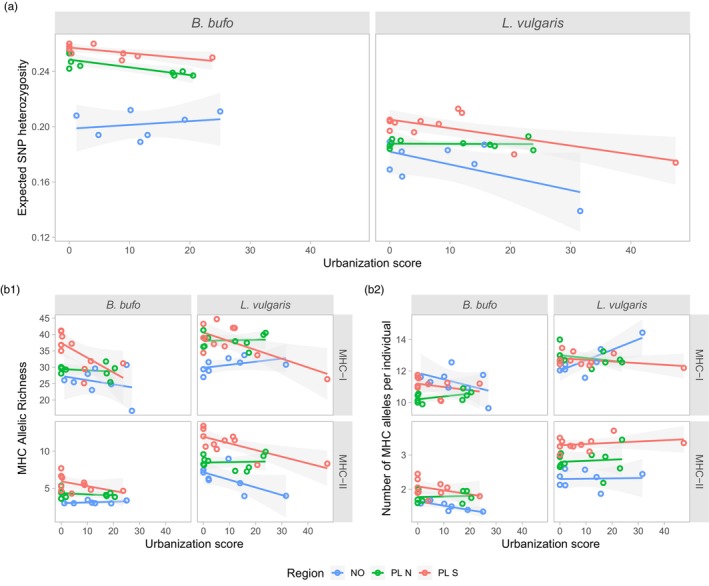
The relationship between measures of diversity in RADseq SNPs (a), major histocompatibility complex (MHC) (b1, b2) and urbanization scores. The trend lines are simple linear regression lines with associated confidence bands.

The effect of urbanization, geographic region, and their interaction on the genetic diversity was tested using two‐way ANOVA (Table 2). In the case of the overall, genome‐wide genetic diversity (measured as expected SNP heterozygosity) no significant interaction between urbanization and region was detected for either species (*B. bufo*, *p* = 0.101, *L. vulgaris*, *p* = 0.114). No effect of urbanization was detected in *B. bufo* (*p* = 0.37), while genome‐wide genetic diversity decreased with increasing urbanization score in *L. vulgaris* (*p* = 0.007, Figure 3a). The effect of region was significant in both species (*B. bufo p* = 7e‐10, *L. vulgaris p* = 6e‐4), with the lowest genomic variation in Norway (Figure 3a). Moran's I test showed no spatial autocorrelation in the residuals of nine out of 10 models, the only exception being the model testing the effect of urbanization on MHC II allelic richness in *B. bufo* (see Table 2).

**TABLE 2:** The effect of urbanization on genetic diversity.

Species	Response variable		Residual	Urbanization score			Region			Urb × Reg			Moran's *I* test		
	Marker	Measure	df	df	*F*	*p*	df	*F*	*p*	df	*F*	*p*	Observed	Expected	*p*
*Bufo bufo*	MHC‐I	AR	21	1	0.69	0.42	2	8.94	0.0016**	2	1.97	0.16	−0.132	−0.217	0.297
		NAllind	21	1	2.99	0.10	2	7.22	0.0041**	2	1.79	0.19	−0.089	−0.217	0.211
	MHC‐II	AR	20	1	0.07	0.79	2	14.43	0.0001***	2	1.29	0.30	0.304	−0.217	0.001***
		NAllind	20	1	1.96	0.18	2	6.14	0.008**	2	1.20	0.32	−0.311	−0.217	0.722
	SNP	Hs	20	1	0.83	0.37	2	72.13	7.00e‐10***	2	2.58	0.10	−0.298	−0.217	0.695
*Lissotriton vulgaris*	MHC‐I	AR	23	1	0.84	0.37	2	18.31	2.00e‐05***	2	5.51	0.011*	−0.208	−0.11	0.733
		NAllind	23	1	15.90	0.0006***	2	5.48	0.011*	2	8.85	0.0014**	−0.24	−0.11	0.796
	MHC‐II	AR	23	1	6.37	0.019*	2	25.88	1.00e‐06***	2	2.50	0.10	−0.218	−0.11	0.755
		NAllind	23	1	0.01	0.92	2	17.37	3.00e‐05***	2	0.03	0.97	−0.113	−0.11	0.508
	SNP	Hs	23	1	8.72	0.007**	2	10.35	0.0006***	2	2.39	0.11	−0.088	−0.11	0.445

*Note*: ANOVA (type III SS) results for linear models testing the effect of urbanization score, region, and their interaction on various measures of genetic variation for genome‐wide SNP, MHC‐I, and MHC‐II markers.

Abbreviations: AR, allelic richness (standardized to the sample size of 8); Hs, expected heterozygosity; NAllind, mean number of alleles per individual.

*p < 0.05, **p < 0.01, ***p < 0.001.

MHC diversity was measured as allelic richness (AR; standardized to the sample size of 8) and the mean number of alleles per individual (NAllind). In *B. bufo* we did not detect any effect of urbanization or urbanization × region interaction on either measure of MHC diversity. In *L. vulgaris*, the AR decreased with urbanization only in MHC‐II (*p* = 0.019), while there was significant urbanization × region interaction in MHC‐I (*p* = 0.011). For both species and MHC classes, the effect of region was highly significant, with the lowest AR in Norway (Table 2, Figure 3b1). In the case of NAllind the effect of urbanization was significant only in *L. vulgaris* MHC‐I (*p* = 0.0006), for which, however, the interaction between urbanization and region was also significant (*p* = 0.0014). The significant interaction suggests that the response differs between regions, with NAllind, surprisingly, increasing with urbanization in Norway but decreasing in both PL N and PL S (Table 2, Figure 3b2). The effect of region on NAllind was significant in all cases (Table 2).

### 3.3 Associations between genetic variants and urbanization

Only three of 33 tested MHC alleles showed an association with urbanization scores after Bonferroni correction: for *B. bufo* MHC‐I 014 and for *L. vulgaris* MHC‐I 0001 and 0010 (Table S1). However, visual inspection of the relationship between urbanization score and allele frequencies (Figure S4) shows that it was not consistent across regions. For *L. vulgaris* MHC‐I 0001 allele the urbanization score × region interaction was significant following Bonferroni correction, while it was significant at the nominal *p* level for the other two alleles (Table S1).

**FIGURE 4:**
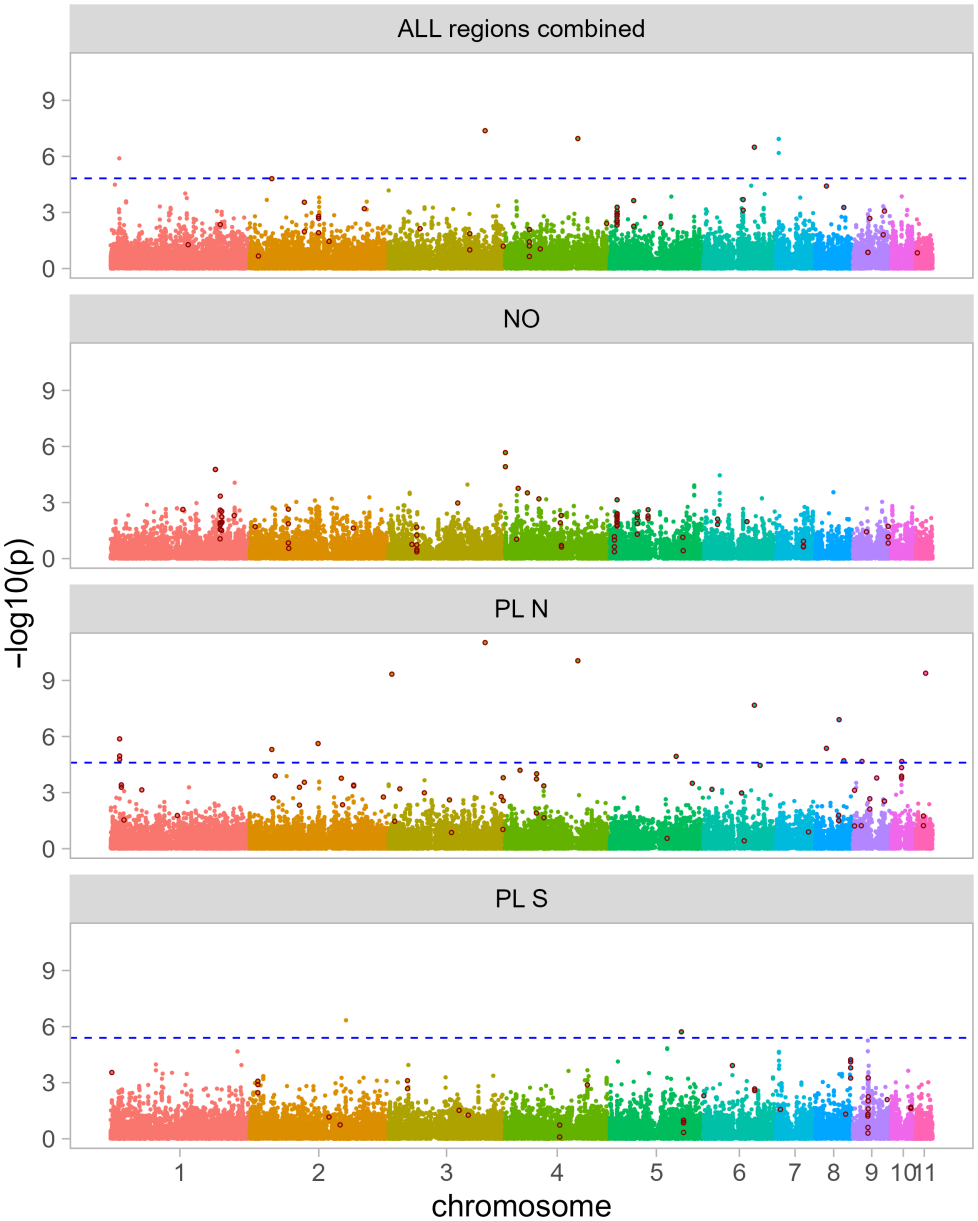
Genomic scans for SNPs associated with urbanization in *Bufo bufo*. Both Latent Factors Mixed Models (LFMM) and BayPass analyses were performed for the entire dataset (all regions combined) and for each region separately (NO, PL N, PL S). *p*‐values from LFMM analysis are presented as dots color‐filled according to chromosome, the dashed blue line indicates the false discovery rate (FDR) threshold of 0.05; as in the region NO no SNP was significant following the FDR procedure, the threshold line was not plotted. The deep red outlined circles are SNPs identified as significant by BayPass.

The GEA analyses in *B. bufo* used 36,695 SNPs with MAF ≥ 0.05. In LFMM 8 SNPs were significant at FDR 0.05 (Figure 4). The associations were not replicated between geographic regions, as no SNPs were significant in NO and significant SNPs did not overlap between PL N and PL S (Table 3). Plots for separate and combined regions indicate that the significant signal at the level of the entire dataset was driven by particular geographic regions. This was further corroborated by a lack of overlap between the slightly genetically differentiated PL N and PL S (Figure S4). Note, however, that some SNPs significant in one dataset could not be tested in others because of insufficient polymorphism (Table 3). The BayPass analysis identified 47 SNPs associated with urbanization, but again the associations were not repeated between regions, as not a single SNP was identified as associated with urbanization in more than one region (Table 3). The detected associations were thus entirely region‐specific and the overall signal was driven by particular regions (Table 3, Figures 4 and S5). However, within regional datasets, there was a highly significant overlap between the methods in all datasets that had any SNPs significant in both methods (all *p* < 2e‐16, Table 3).

**TABLE 3:** Overlap of urbanization candidates between datasets.

Species	Level	Method	Dataset A	Dataset B	Tested in both datasets					Tested in any dataset	
					Nonsig in both	Sig A only	Sig B only	Sig A & B	*p*	Sig A	Sig B
*Bufo bufo*	SNP	LFMM	ALL	NO	24,924	0	0	0	–	8	0
*B. bufo*	SNP	LFMM	ALL	PL N	29,445	0	7	3	0	8	16
*B. bufo*	SNP	LFMM	ALL	PL S	30,647	0	1	0	–	8	2
*B. bufo*	SNP	LFMM	NO	PL N	18,760	0	0	0	–	0	16
*B. bufo*	SNP	LFMM	NO	PL S	19,579	0	1	0	–	0	2
*B. bufo*	SNP	LFMM	PL N	PL S	28,609	4	2	0	1	16	2
*B. bufo*	SNP	BayPass	ALL	NO	24,839	13	62	10	0	47	76
*B. bufo*	SNP	BayPass	ALL	PL N	29,370	34	43	8	0	47	70
*B. bufo*	SNP	BayPass	ALL	PL S	30,584	35	26	3	0	47	39
*B. bufo*	SNP	BayPass	NO	PL N	18,678	56	26	0	1	76	70
*B. bufo*	SNP	BayPass	NO	PL S	19,508	58	14	0	1	76	39
*B. bufo*	SNP	BayPass	PL N	PL S	28,553	39	23	0	1	70	39
*Lissotriton vulgaris*	SNP	LFMM	ALL	NO	271,134	473	39	0	1	1986	39
*L. vulgaris*	SNP	LFMM	ALL	PL N	308,448	1277	2	0	1	1986	2
*L. vulgaris*	SNP	LFMM	ALL	PL S	335,056	1751	70	226	0	1986	300
*L. vulgaris*	SNP	LFMM	NO	PL N	179,332	16	1	0	1	39	2
*L. vulgaris*	SNP	LFMM	NO	PL S	199,472	22	85	0	1	39	300
*L. vulgaris*	SNP	LFMM	PL N	PL S	284,301	2	195	0	1	2	300
*L. vulgaris*	SNP	BayPass	ALL	NO	269,473	426	1706	41	0	883	1791
*L. vulgaris*	SNP	BayPass	ALL	PL N	308,513	530	521	163	0	883	685
*L. vulgaris*	SNP	BayPass	ALL	PL S	334,438	557	1914	194	0	883	2125
*L. vulgaris*	SNP	BayPass	NO	PL N	177,998	1023	322	6	0.0081	1791	685
*L. vulgaris*	SNP	BayPass	NO	PL S	197,326	1282	963	8	0.62	1791	2125
*L. vulgaris*	SNP	BayPass	PL N	PL S	282,334	582	1579	3	1	685	2125
*L. vulgaris*	RAD locus	LFMM	ALL	NO	36,060	576	18	0	1	785	18
*L. vulgaris*	RAD locus	LFMM	ALL	PL N	39,938	725	1	1	0.013	785	2
*L. vulgaris*	RAD locus	LFMM	ALL	PL S	41,470	670	25	115	0	785	141
*L. vulgaris*	RAD locus	LFMM	NO	PL N	32,947	15	2	0	1	18	2
*L. vulgaris*	RAD locus	LFMM	NO	PL S	34,125	17	96	0	1	18	141
*L. vulgaris*	RAD locus	LFMM	PL N	PL S	39,262	2	130	0	1	2	141
*L. vulgaris*	RAD locus	BayPass	ALL	NO	35,888	293	445	28	0	394	478
*L. vulgaris*	RAD locus	BayPass	ALL	PL N	40,153	295	155	62	0	394	217
*L. vulgaris*	RAD locus	BayPass	ALL	PL S	41,344	283	562	91	0	394	655
*L. vulgaris*	RAD locus	BayPass	NO	PL N	32,386	415	161	2	1	478	217
*L. vulgaris*	RAD locus	BayPass	NO	PL S	33,305	437	489	7	0.98	478	655
*L. vulgaris*	RAD locus	BayPass	PL N	PL S	38,594	205	591	4	0.85	217	655

*Note*: Level indicates whether testing was performed at the level of individual SNPs or at the level of RAD loci; Dataset: ALL, all populations analyzed together; NO, only Norway, PL N, only northern Poland; PL S, only southern Poland; *p* – significance of the overlap of candidates between datasets, “–” indicates that the test could not be performed, and “0” indicates *p* < 2ec‐16.

**FIGURE 5:**
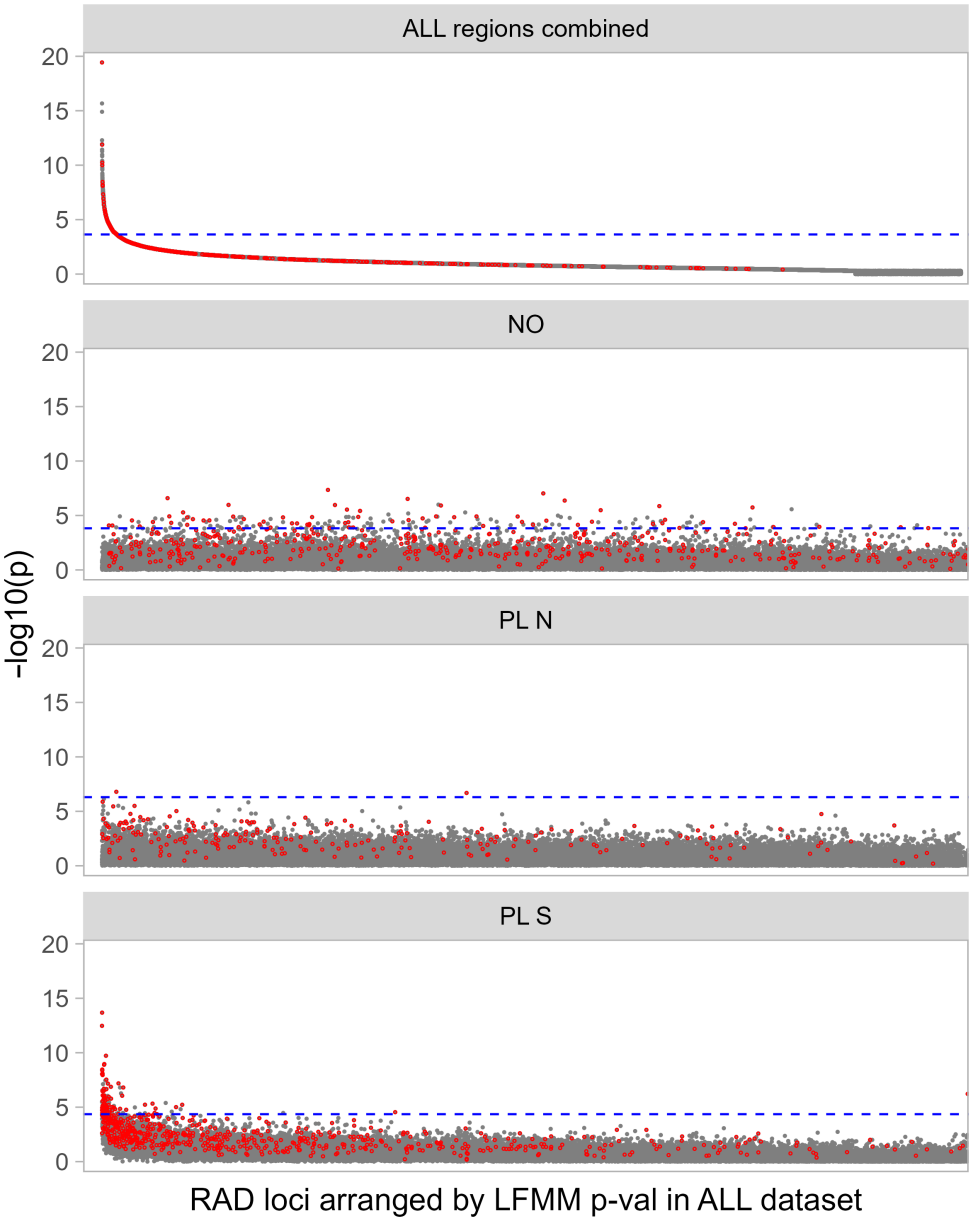
Genomic scans for SNPs associated with urbanization in *Lissotriton vulgaris*. Both Latent Factors Mixed Models (LFMM) and BayPass analyses were performed for the entire dataset (all regions combined) and for each region separately (NO, PL N, PL S). The minimum per RAD locus *p*‐values from LFMM analysis are presented as grey dots, arranged in all panels according to the *p*‐value in the entire dataset. The dashed blue line indicates the false discovery rate (FDR) threshold of 0.05. The red outlined circles are SNPs identified as significant by BayPass.

In *L. vulgaris*, we excluded Lv_NO_9 due to the extremely low genetic diversity and extremely high divergence identified for this population, which could bias the results. The GEA analyses in *L. vulgaris* used 428,319 SNPs with MAF ≥0.05. The results were qualitatively similar to those in *B. bufo* (Figures 5 and S6). In LFMM there were as many as 1986 SNPs significant for the entire dataset, however none were shared between any two regions (Table 3). In BayPass, a total of 883 SNPs were identified as associated with urbanization at the level of the entire dataset. Interestingly, the number of SNPs identified by BayPass as associated with urbanization in NO (1791) and PL S (2125) was much higher than identified in the entire dataset. We observed some overlap between regions in BayPass results, as six SNPs overlapped between NO and PL N, eight between NO and PL S, and three between PL N and PL S. However, only the overlap between NO and PL N was significant (*p* = 0.008). As there is currently no reference genome available for *L. vulgaris*, we aggregated SNP data by RAD locus. Both LFMM and BayPass results at the locus level were very similar to those for the SNP level analysis, showing no overlap of candidates between regions (Table 3, Figures 5 and S6), as even the overlap between NO and PL N in BayPass was insignificant (Table 3). Also, in *L. vulgaris* there was a highly significant overlap of candidates identified by both methods for the same datasets (all *p* < 2e‐16, Table 4).

**TABLE 4:** Overlap between urbanization candidates identified by Latent Factors Mixed Models (LFMM) and BayPass for the same dataset.

Species	Level	Dataset	Nonsig in both	Sig LFMM only	Sig BayPass only	Sig LFMM & BayPass	*p*
*Bufo bufo*	SNP	ALL	36,493	5	44	3	0
*B. bufo*	SNP	NO	26,830	0	76	0	–
*B. bufo*	SNP	PL N	30,380	0	54	16	0
*B. bufo*	SNP	PL S	32,159	1	38	1	0
*Lissotriton vulgaris*	SNP	ALL	425,534	1902	799	84	0
*L. vulgaris*	SNP	NO	278,495	8	1760	31	0
*L. vulgaris*	SNP	PL N	310,152	0	683	2	0
*L. vulgaris*	SNP	PL S	336,761	140	1965	160	0
*L. vulgaris*	RAD locus	ALL	44,328	728	337	57	0
*L. vulgaris*	RAD locus	NO	36,457	4	464	14	0
*L. vulgaris*	RAD locus	PL N	40,510	0	215	2	0
*L. vulgaris*	RAD locus	PL S	41,668	63	577	78	0

*Note*: Level indicates whether testing was performed at the level of individual SNPs or at the level of RAD loci; Dataset: ALL, all populations analyzed together; NO, only Norway; PL N, only northern Poland; PL S, only southern Poland. *p* – significance of the overlap of candidates between datasets, “–” indicates that the test could not be performed, and “0” indicates *p* < 2e‐16.

### 3.4 Genes associated with urbanization candidates

Since almost all candidates were region‐specific, we combined all candidates within species into a single category for the analysis of candidate‐associated genes. Overall, considering all datasets and both GEA methods, there were 219 and 7040 SNPs associated with urbanization in *B. bufo* and *L. vulgaris*, respectively. Such a defined candidate set is likely to contain many false positives. However, if adaptation to urbanization is polygenic and region‐specific, then many relevant SNPs will show only a weak signal, likely to pass the significance threshold only in some datasets. If, however, urbanization associated genes are involved in specific biological processes or functions, the aggregate signal may be revealed by gene set analysis. In *L. vulgaris*, the analysis was performed at the level of RAD loci. Only 814 out of 45,855 tested loci (1.8%) were unambiguously mapped to the *P. waltl* genome; 35 out of 2261 (1.5%) loci containing candidate SNPs were mapped. The proportion of urbanization candidates within CDS (*B. bufo* 1.8%, *L. vulgaris* 28.6%) or associated with genes (*B. bufo* 42%, *L. vulgaris* 60%) was not elevated in either species (chi‐squared test, *B. bufo* CDS *p* = 0.44, genes *p* = 0.34, *L. vulgaris* CDS *p* = 0.91, genes *p* = 0.67).

Of 4503 *B. bufo* and 509 *L. vulgaris* genes associated with markers tested in GEA, 3547 and 407 had GO terms assigned, respectively. None of 44 *B. bufo* and 15 *L. vulgaris* GO‐assigned genes associated with urbanization candidates were shared between species (Table S2). Among these genes, three biological process (BP), two cellular component (CC), and one molecular function (MF) categories were enriched at the *p* value <0.01 threshold in *B. bufo*, and 11 BP, two CC, and one MF were enriched in *L. vulgaris* (Table S3). None of the enriched categories were shared between the species.

## 4 DISCUSSION

We investigated genomic signatures of urbanization in two amphibian species present in three widely separated geographic regions of northcentral Europe by comparing allele frequencies at tens (*B. bufo*) or hundreds (*L. vulgaris*) of thousands of SNPs. We also examined variation in MHC class I and class II loci across the rural–urban gradient for both species, as these genes should be under selection in relation to pathogen pressure. Our analyses showed that most of the genetic differentiation could be attributed to regional (latitudinal) effects, most likely linked to historical biogeography. We did not find any association between genetic differentiation among populations and the level of urbanization in either species. However, our analyses show that urban newts, but not toads, have lower within population levels of genetic diversity, suggesting higher susceptibility to the negative effects of urbanization in the former. Moreover, although GEA analyses revealed numerous candidate SNPs linked to urbanization in both species, we found a marked lack of repeatability between the geographic regions, suggesting a complex and multifaceted response to natural selection elicited by life in the city.

### 4.1 Urbanization does not lead to higher population differentiation

For species with a patchy population structure such as pond‐breeding amphibians, urbanization may diminish or preclude connectivity among populations, lowering population viability and ultimately leading to local extinction. Overall, we did not observe elevated differentiation or higher levels of population‐specific genetic drift for populations of two amphibian species in the urbanized regions of three rural–urban gradients in northcentral Europe. This result cannot be attributed to a lack of resolution of the employed molecular markers, as our RADseq protocol generated thousands of SNPs spread across the genomes of these two species. We also targeted variable MHC class I and II, genes shown to rapidly respond to novel environmental selection pressures (Minias, 2023; Phillips et al., 2018). Thus, our primary conclusion is that the studied amphibian populations lack the population genetic signs of spatial isolation and cessation of gene flow predicted for many urban dwelling organisms (Johnson & Munshi‐South, 2017). Despite being particularly vulnerable to habitat fragmentation (Cushman, 2006), our current results and previous work (Schmidt & Garroway, 2021 and references therein) suggest that habitat loss does not invariably lead to adverse genetic effects in amphibian populations, at least not in the short‐term. Several factors may explain this result.

As pointed out by others (Miles et al., 2019; Rivkin et al., 2019; Schmidt & Garroway, 2021) the effects of urbanization may be species‐ and context‐specific. The same landscape elements, whether human‐made or natural, can have disparate effects on patterns of gene flow for different amphibian species (e.g., Antunes, Figueiredo‐Vázquez, et al., 2023; Homola, Loftin, & Kinnison, 2019). Although the effect of landscape features on patterns of gene flow was beyond the scope of this study, we found a broadly similar effect of urbanization on both studied species. Common toads and smooth newts are generalists that are tolerant to a wide range of habitats (Juszczyk, 1987; Speybroeck et al., 2016). Both species have rather high levels of site fidelity but can move more than 1 km from natal ponds, regularly and exceptionally in the case of toads and newts, respectively (Beebee & Griffiths, 2000; Kovar et al., 2009; Schmidt et al., 2006; Sinsch, 1988). One possible explanation for the lack of influence of urbanization on population differentiation is that the landcover types in the studied areas are relatively easy to move through and therefore may not be predictive of functional disconnectivity across a larger landscape for either of the species. For instance, vegetated urban corridors or waterways may allow for weak to moderate gene flow between urban populations of toads and newts in our study areas, countering the genetic effects of population isolation.

Another possible explanation for our results may be a time lag between urbanization and its effect on population structure of the newt and toad populations. Time lags, that is, the number of generations between landscape perturbations and a discernible population genetic response, could be particularly severe in urban areas because a rapid pace of urbanization may prevent genetic parameters of populations from approaching new equilibrium values (Epps & Keyghobadi, 2015). However, recent work has shown that *L. vulgaris* rapidly responds to changes in landscape structure with no evidence for time lags (Antunes, Dudek, et al., 2023), and retains high connectivity in modified habitat, for example, forest edges (Antunes, Figueiredo‐Vázquez, et al., 2023). In city‐dwelling salamanders, Lourenço et al. (2017) did not find an effect of demographic history and time since isolation on genetic diversity within populations. On the other hand, anthropogenic landscapes decrease connectivity and effective population sizes in *L. vulgaris* (Antunes, Figueiredo‐Vázquez, et al., 2023). Out of all urban populations of both studied species, only one Norwegian smooth newt population (Lv_NO_9) showed differentiation attributable to extremely strong genetic drift possibly associated with a founder event as this population inhabited a highly urbanized area.

### 4.2 Inconsistent influence of urbanization on genetic diversity

Urbanization had different impacts on levels of genetic diversity within populations for smooth newts and common toads. We did not find any effect of urbanization on genome‐wide or MHC diversity in *B. bufo*. However, genome‐wide genetic diversity and allelic richness in MHC‐I decreased with increasing urbanization level in *L. vulgaris*. Our results suggest that urban smooth newt populations have gone through recent bottlenecks, although we cannot rule out that these predate the expansion of the cities. On the other hand, the lack of genetic evidence for recent bottlenecks in the studied toad populations suggest that they have persisted in the local landscape since before the onset of urbanization. The contrasting responses of the two studied species imply that within cities, toads have larger effective population sizes and higher connectivity among populations than newts. *Bufo bufo* occurs in many semi‐natural spaces such as parks and community gardens, suggesting that it is less strongly affected by urban fragmentation. *Bufo bufo* exhibit explosive breeding in which most adult individuals congregate at ponds for a short but intense breeding period. This mating strategy may increase local effective population sizes in comparison to species with a protracted breeding period, such as newts. Unexpectedly high values of genetic diversity within populations were also found in salamanders inhabiting putatively isolated, small patches of suitable habitat in the city of Oviedo, despite high differentiation between populations (Lourenço et al., 2017).

The effects of urbanization on genetic variation were found to be consistent in some vertebrate groups, for example, mammals, but not birds or amphibians (Schmidt et al., 2020; Schmidt & Garroway, 2021), suggesting that the responses have group‐ as well as species‐specific determinants. For instance, birds are more vagile than mammals or amphibians and may therefore be less sensitive to fragmentation. Interestingly, Schmidt and Garroway (2022) showed a parallel negative influence of urbanization across vertebrates (including amphibians) within cities. Our replicated design involving two common and relatively abundant city‐dwelling amphibians adds evidence to the contrary, that is, that the effects of urbanization on genetic variation are indeed species‐specific. Nonetheless, in areas of fast‐paced urban growth, species less susceptible to the onset of genetic erosion in cities, such as common toads, may become extirpated before the effects of urbanization become detectable at the genetic level.

### 4.3 Regional differentiation prevails

We found evidence for a strong regional signal of genetic differentiation and levels of genetic diversity in genome‐wide SNP and MHC variation in both amphibian species. All three regional groups were differentiated from one another, but the Norwegian populations have experienced more population‐specific drift compared to the Polish sites. Post‐glacial colonization history could account for the general differences between regions, with the more northerly Norwegian populations most affected by historical founder events. Both species are thought to have colonized northcentral Europe from more southerly Pleistocene refugia (Babik et al., 2005; Garcia‐Porta et al., 2012; Pabijan et al., 2015; Recuero et al., 2012). Interestingly, we found some evidence for substructure in *B. bufo* in Norway and northern Poland, areas that were previously shown to have remarkably little genetic differentiation using microsatellites and mtDNA (Brede & Beebee, 2006; Garcia‐Porta et al., 2012; Recuero et al., 2012), implying that the use of more informative molecular markers such as large SNP datasets could be used to reveal the historical biogeography of this species in northern latitudes.

### 4.4 Inconsistent genetic‐environment associations among regions

Both applied GEA methods identified a number of SNPs associated with urbanization in each species. However, the most remarkable feature of these urbanization candidates was their regional specificity. While the sets of candidates identified by BayPass and LFMM overlapped considerably for the same dataset, there was virtually no overlap between candidates identified in different geographic regions, and the signal of association with urbanization in the entire dataset was driven by single regions. There may be several explanations for the lack of repeatability. First, adaptation to urbanization may not occur or be detectable with our study design, and both methods may have picked up the same artifactual signal, for example, due to insufficient correction for geographic structuring. A simple lack of adaptation cannot be ruled out, but other studies of amphibians have suggested rapid adaptation to human‐modified environments, such as the vicinity of roads (Brady, 2012; Hopkins et al., 2013) and urban areas (Homola, Loftin, Cammen, et al., 2019). Second, the genomic basis of adaptation may indeed differ between geographic regions. This, in turn, may result from differences in the strength and mode of selection imposed by the urban environment due to inherent differences between cities (Santangelo et al., 2020). However, it is also possible that similar phenotypic responses have different genomic bases. The probability of this scenario depends on the genomic architecture of the responding traits (Sella & Barton, 2019), with reduced likelihood of genetic parallelism despite phenotypic parallelism for highly polygenic traits (Barghi et al., 2020). Finally, regional differences in response to urbanization could also result from geographically variable interactions between urbanization and other environmental factors such as temperature that varies with latitude (Palomar et al., 2023).

Typically, both parallel and region‐specific signals of adaptation to urbanization are detected in genomic scans (Reid et al., 2016; Salmón et al., 2021), although some studies have found little evidence for a parallel genomic response to urbanization (Babik et al., 2023; Caizergues et al., 2022; Mueller et al., 2020). A replicable signal of adaptation to urbanization has been detected in another amphibian, the wood frog (Homola, Loftin, Cammen, et al., 2019).

## 5 CONCLUSIONS

Understanding the origins of evolutionary change in city‐dwelling species is important for conservation and natural resource management in the urban network. Counter to our expectations, urbanization does not seem to have resulted in increased differentiation of urban amphibian populations in our study areas, compared to rural counterparts, suggesting that gene flow between them is relatively unaffected. The landscape elements that maintain weak or moderate connectivity among these populations have yet to be identified, although our results suggest that they may be common to all three of the studied cities. A consistent reduction of genetic diversity at urban sites was found in only one of two studied species, suggesting that newts are more prone to urban bottlenecks (through founder events or sharp demographic declines) than toads. We conclude that, overall, the extent of urbanization has not yet reached levels significantly affecting nonadaptive evolutionary processes for newt and toad populations in the studied areas. Our replicated design involving two common and relatively abundant species supports species‐specific responses to urbanization in amphibians (Schmidt & Garroway, 2021). Our data also indicate that genetic variants associated with an urban environment, which can be interpreted as genomic beacons of adaptation, occur locally and are not subject to parallel evolution. The effect of urbanization may depend on its interactions and synergies with other environmental factors and hence, the adaptative response to urban life in the examined amphibians seems to be multi‐faceted and the consequence of city‐specific urban features. Our results do not preclude a deleterious effect of urbanization on amphibians in the studied cities. Indeed, we found diminished levels of genome‐wide variation in newts. Moreover, we suspect that due to the rapid pace of city expansion occurring particularly in the Polish cities, urban amphibian populations may become extirpated before urban‐induced genetic effects become detectable.

## CONFLICT OF INTEREST STATEMENT

The author declares no conflict of interest.

## Supporting information


Table S1.
Table S2.
Table S3.

Figure S1.
Figure S2.
Figure S3.
Figure S4.
Figure S5.
Figure S6.

## Data Availability

The data that support the findings of this study are openly available in NCBI Sequence Read Archive at https://www.ncbi.nlm.nih.gov/sra, project numbers PRJNA1080208 and PRJNA1080284 (raw sequencing reads) and in Dryad at doi:10.5061/dryad.9ghx3ffr2 (genotypes and associated metadata).
