# Supplementary material for: Limited evidence for genetic differentiation or adaptation in two amphibian species across replicated rural–urban gradients
Source: Evol Appl. 2024 Jun 3;17(6):e13700. doi: 10.1111/eva.13700 (PMC11146147; doi:10.1111/eva.13700)
Supplement: Supplementary file 2 — Figure S1. Figure S2. Figure S3. Figure S4. Figure S5. Figure S6. [file EVA-17-e13700-s001.docx]

# Limited evidence for genetic differentiation or adaptation in two amphibian species across replicated rural-urban gradients

# Supplementary Materials

## Supplementary Tables

Are in a separate Excel workbook

## Supplementary Figures

**Fig. S1. MHC allele frequencies in regions.** Fraction of individuals carrying a particular allele in each region is plotted.

**Fig. S2. Multidimensional scaling of MHC pairwise *F*_ST_ matrices between populations.**

**Fig. S3. The distribution of urbanization scores.** NO, PL N and PLS indicate the studied geographic regions, while points indicate localities.

**Fig. S4. Frequencies of the three MHC alleles significantly associated with urbanization.**


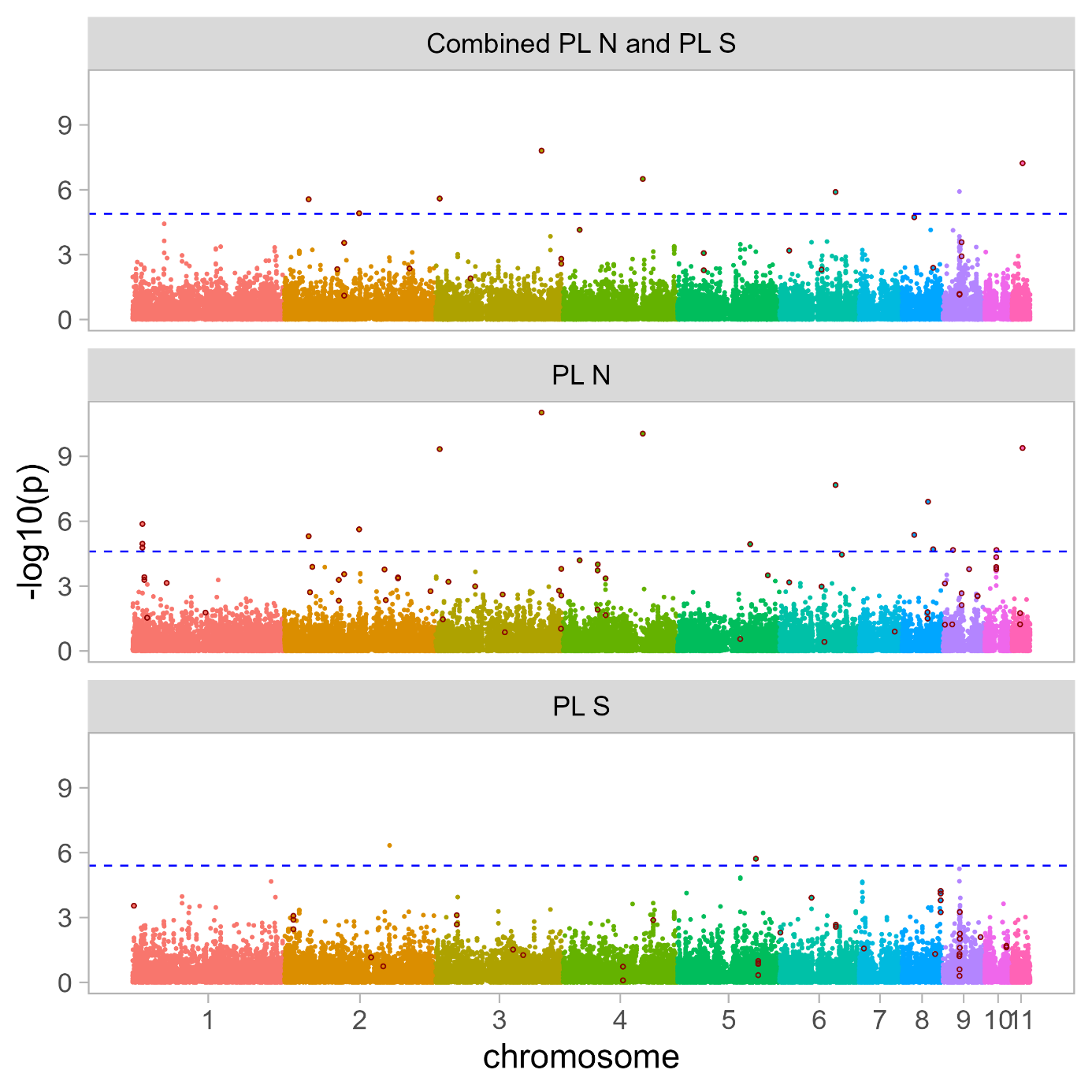


**Fig. S5. Genomic scans for SNPs associated with urbanization in *B. bufo* in Polish populations.** Both LFMM and BayPass analyses were performed for the entire Polish dataset (Combined PL N and PL S) and for each region separately (PL N, PL S). *P*-values from LFMM analysis are presented as dots colour-filled according to chromosome, the dashed blue line indicates the false discovery rate (FDR) threshold of 0.05. The deep red outlined circles are SNPs identified as significant by BayPass.


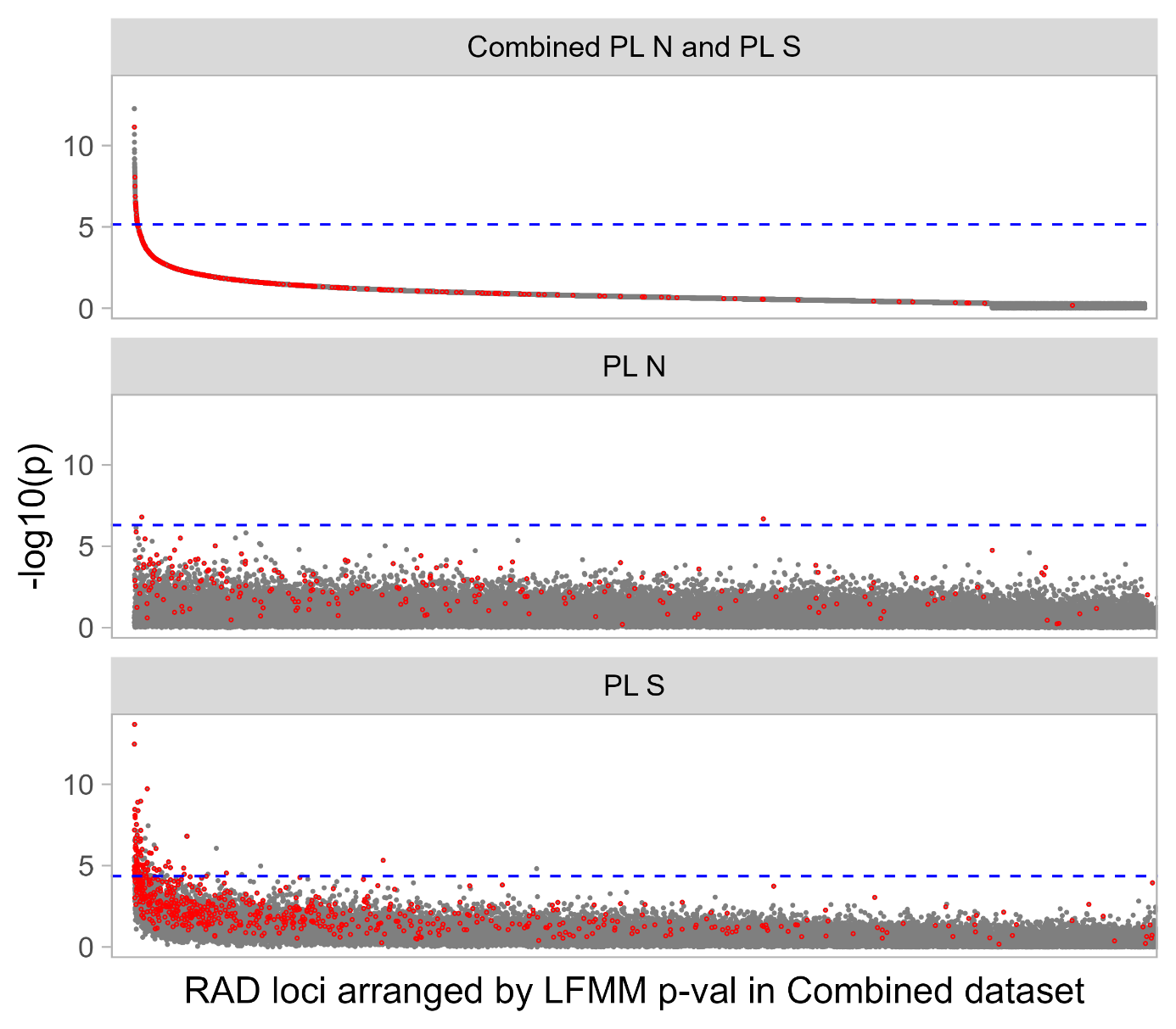


**Fig. S6. Genomic scans for SNPs associated with urbanization in *L. vulgaris* in Polish populations.** Both LFMM and BayPass analyses were performed for the entire Polish dataset Combined PL N and PL S) and for each region separately (PL N, PL S). The minimum per RAD locus *P*-values from LFMM analysis are presented as grey dots, arranged in all panels according to the P-value in the entire dataset. The dashed blue line indicates the false discovery rate (FDR) threshold of 0.05. The red outlined circles are SNPs identified as significant by BayPass.
